# Interleukin-13 promotes cellular senescence through inducing mitochondrial dysfunction in IgG4-related sialadenitis

**DOI:** 10.1038/s41368-022-00180-6

**Published:** 2022-06-20

**Authors:** Mengqi Zhu, Sainan Min, Xiangdi Mao, Yuan Zhou, Yan Zhang, Wei Li, Li Li, Liling Wu, Xin Cong, Guangyan Yu

**Affiliations:** 1 Department of Oral and Maxillofacial Surgery, Peking University School and Hospital of Stomatology & National Center of Stomatology & National Clinical Research Center for Oral Diseases & National Engineering Research Center of Oral Biomaterials and Digital Medical Devices, Beijing, China; 2 Department of Physiology and Pathophysiology, Peking University School of Basic Medical Sciences, Key Laboratory of Molecular Cardiovascular Sciences, Ministry of Education, and Beijing Key Laboratory of Cardiovascular Receptors Research, Beijing, China; 3 Department of Biomedical Informatics, Peking University School of Basic Medical Sciences, Key Laboratory of Molecular Cardiovascular Sciences, Ministry of Education, Beijing, China

**Keywords:** Salivary gland diseases, Mechanisms of disease

## Abstract

Immunoglobulin G4-related sialadenitis (IgG4-RS) is an immune-mediated fibro-inflammatory disease and the pathogenesis is still not fully understood. The aim of this study was to explore the role and mechanism of interleukin-13 (IL-13) in the cellular senescence during the progress of IgG4-RS. We found that the expression of IL-13 and IL-13 receptor α1 (IL-13Rα1) as well as the number of senescent cells were significantly higher in the submandibular glands (SMGs) of IgG4-RS patients. IL-13 directly induced senescence as shown by the elevated activity of senescence-associated β-galactosidase (SA-β-gal), the decreased cell proliferation, and the upregulation of senescence markers (p53 and p16) and senescence-associated secretory phenotype (SASP) factors (IL-1β and IL-6) in SMG-C6 cells. Mechanistically, IL-13 increased the level of phosphorylated signal transducer and activator of transcription 6 (p-STAT6) and mitochondrial-reactive oxygen species (mtROS), while decreased the mitochondrial membrane potential, ATP level, and the expression and activity of superoxide dismutase 2 (SOD2). Notably, the IL-13-induced cellular senescence and mitochondrial dysfunction could be inhibited by pretreatment with either STAT6 inhibitor AS1517499 or mitochondria-targeted ROS scavenger MitoTEMPO. Moreover, IL-13 increased the interaction between p-STAT6 and cAMP-response element binding protein (CREB)-binding protein (CBP) and decreased the transcriptional activity of CREB on SOD2. Taken together, our findings revealed a critical role of IL-13 in the induction of salivary gland epithelial cell senescence through the elevated mitochondrial oxidative stress in a STAT6–CREB–SOD2-dependent pathway in IgG4-RS.

## Introduction

Immunoglobulin G4-related disease (IgG4-RD) is a systemic disease characterized by elevated serum IgG4 concentration, multi-organ inflammation, infiltration of IgG4^+^ plasma cells, and storiform fibrosis in various organs, including the liver, kidney, salivary gland, pancreas, and lung.^1^ Salivary gland is one of the most commonly affected organs, which is called IgG4-related sialadenitis (IgG4-RS).^2^ The main symptoms of IgG4-RS are the painless enlargement of the involved salivary glands and varying degrees of hyposecretion, both of which seriously threaten patient’s quality of life.^3^ The pathogenesis of IgG4-RS is still not clear and effective treatments are limited. Recent studies have shown that unlike other autoimmune disorders such as Sjögren’s syndrome, which are mainly related to the dysfunction of T helper 1 (Th1) and/or Th17 subset, IgG4-RD is considered to be dominantly caused by Th2 or a combination of Th2 and regulatory T cells.^1^ The activated Th2 cells produce an inflammatory cytokine milieu that promotes the maturation from B cells into plasma cells and the isotype switch towards IgG4.^4^ Among the Th2-secreted cytokines, interleukin-13 (IL-13) plays a crucial role in the progression of allergic diseases and has been found to be significantly elevated in the serum and affected tissues of the patients suffering from many autoimmune diseases, such as Sjögren’s syndrome, rheumatoid arthritis, and systemic sclerosis.^5^ Notably, the number of IL-13-positive cells in the submandibular glands (SMGs) of IgG4-RS patients is much higher than that of submandibular sialolithiasis and normal controls.^6^ These data indicated that IL-13 might be a specific cytokine responsible for the occurrence of IgG4-RS. However, the exact function and mechanism of IL-13 in IgG4-RS was poorly understood.

Cellular senescence is referred to a state of irreversibly arrested cell cycle which can be induced by DNA damage, mitochondrial dysfunction, oncogenic activation, telomere erosion, chronic inflammation, and oxidative stress.^7^ The key features of senescent cells include decreased cell proliferation, increased activity of senescence-associated β-galactosidase (SA-β-gal), and high expression of cyclin-dependent kinase inhibitor genes, such as p53, p21, and p16. Another remarkable feature of senescent cells is the transition into the senescence-associated secretory phenotype (SASP) that produces a complex secretome, including IL-1β, IL-6, and transforming growth factor-β1 (TGF-β1).^8,9^ In recent years, much attention has been paid to mitochondrial dysfunction as a pathological factor in cellular senescence. Since mitochondria are not only the primary source of reactive oxygen species (ROS) production but also the main immediate target of ROS, the oxidative stress induced by elevated ROS would then cause mitochondrial dysfunction.^10^ Previous researches have found that human serum IL-13 level is positively associated with age and IL-13 promotes the cellular senescence in IMR90 human embryonic lung fibroblasts and human umbilical vein endothelial cells.^11,12^ Moreover, IL-13 promotes ROS production in human airway epithelial cells and rat hippocampal neurons.^13,14^ But whether IL-13 could induce the cellular senescence in salivary glands through mitochondrial dysfunction and herein participate in IgG4-RS was totally unknown. Hence, this study sought to reveal the specific involvement of IL-13 and underlying mechanism during IgG4-RS, which might enrich our knowledge of the disease and contribute to the development of novel therapeutic approaches for the treatment of IgG4-RS.

## Results

### Senescent epithelial cells increase and the level of IL-13 and IL-13 receptor α1 (IL-13Rα1) elevate in the SMGs of IgG4-RS patients

**Fig. 1:**
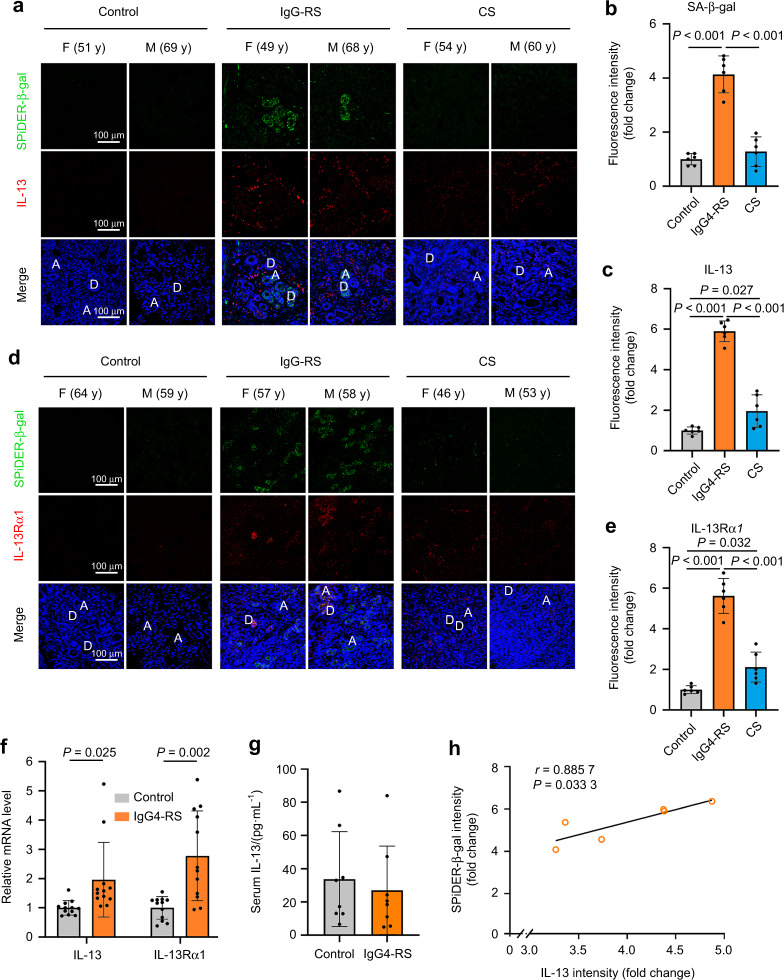
The number of senescent cells and expression of IL-13 and IL-13α1 are significantly increased in the SMGs of IgG4-RS patients. **a**–**e** Immunofluorescence staining (**a**, **d**) and quantitative analysis of SA-β-gal (**b**), IL-13 (**c**), and IL-13Rα1 (**e**) in the SMGs of IgG4-RS patients (*n* = 6), controls (*n* = 6), and CS patients (*n* = 6). A acinus, D duct, M male, F female, y years old. Bars = 100 μm. **f** Quantitative real-time PCR analysis showing the expressions of IL-13 and IL-13Rα1 in the SMGs of IgG4-RS patients (*n* = 12) and controls (*n* = 12). **g** IL-13 level in the serum of IgG4-RS patients (*n* = 8) and controls (*n* = 8) was detected by cytokine antibody array. **h** Correlation between the immunofluorescence staining intensity of IL-13 and the activity of SA-β-gal in the SMGs of IgG4-RS patients (*n* = 6) were analyzed by Spearman test. The significance of differences between groups was analyzed by unpaired Student’s *t*-test (**f**, **g**) or one-way ANOVA followed by Bonferroni’s test (**b**, **c**, **e**). Data are presented as the mean ± SD

To explore whether cellular senescence happened in the SMGs of patients with IgG4-RS, SA-β-gal staining was used. More SA-β-gal positive cells were observed in the residual both acinar and ductal cells in IgG4-RS than that in controls (Fig. 1a, b, d). By contrast, the positive staining was less observed in the samples from patients with chronic sialadenitis (CS), another inflammatory and fibrotic diseases in SMGs, which suggested that the cellular senescence might be a specific phenomenon in IgG4-RS.

Since Th2 lymphocytes and the related secreted cytokines are thought to play a dominant role in the pathogenesis of IgG4-RD, we therefore focused on IL-13 and its canonical receptor IL-13Rα1.^15^ Immunofluorescence staining results revealed that the level of IL-13 (Fig. 1a, c) and IL-13Rα1 (Fig. 1d, e) were markedly elevated in the SMGs of IgG4-RS patients compared with those in controls. IL-13-positive cells were mainly located around the residual acinar and ductal cells as well as within the infiltrated lymphocytic foci. IL-13Rα1-positive cells were mainly located around the residual acinar and ductal cells. In addition, although the staining intensities of IL-13 and IL-13Rα1 in CS patients showed slight increases than those in controls, yet they were still much lower than those in IgG4-RS. In addition, the mRNA expression of IL-13 and IL-13Rα1 were also higher in the SMGs of IgG4-RS patients compared with those in age-matched controls (Fig. 1f). However, no significant change was found in circulating IL-13 level in the serum samples of IgG4-RS patients ((27.06 ± 9.43) pg·mL^−1^) compared with that of controls ((33.79 ± 10.11) pg·mL^−1^) (Fig. 1g). Furthermore, the IL-13 immunofluorescence intensity was positively correlated with SA-β-gal activity in the SMGs of patients with IgG4-RS (Fig. 1h). These results suggested that the salivary gland epithelial cells underwent cellular senescence, which might be closely related with the local IL-13 in the glandular tissues of IgG4-RS.

### IL-13 directly induces cellular senescence in SMG epithelial cells

**Fig. 2:**
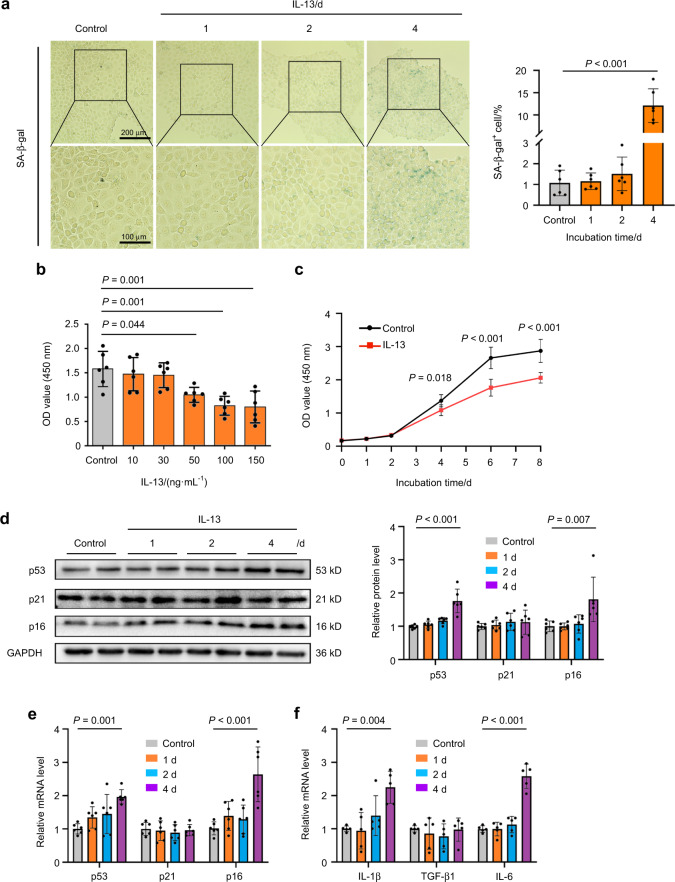
IL-13 promotes cellular senescence in SMG-C6 cells. **a** SA-β-gal staining (left) and quantitative analysis (right) in the SMG-C6 cells treated with IL-13 (50 ng·mL^−1^) for 1, 2, and 4 days (*n* = 6 per group). **b** SMG-C6 cell proliferation ability was evaluated by CCK8 experiment after treatment with different concentrations of IL-13 for 4 days (*n* = 6 per group). **c** SMG-C6 cell proliferation ability was detected by CCK8 experiment after IL-13 (50 ng·mL^−1^) treated for different time points (*n* = 6 per group). **d** Western blot and quantitative analysis showing the protein expression of p53, p21, and p16 in SMG-C6 cell after IL-13 (50 ng·mL^−1^) treatment for 1, 2, and 4 days (*n* = 6 per group). **e**, **f** Quantitative real-time PCR analysis showing the mRNA expression of p53, p21, p16 (**e**) (*n* = 6 per group), IL-1β, TGF-β1, and IL-6 (**f**) (*n* = 5 per group) after IL-13 (50 ng·mL^−1^) treatment for 1, 2, and 4 days in SMG-C6 cells. The significance of differences between groups was analyzed by one-way ANOVA followed by Bonferroni’s test. Data are presented as the mean ± SD

To determine whether the elevated IL-13 in the SMGs of IgG4-RS was involved in the cellular senescence, the cultured SMG-C6 cells were directly treated with IL-13 (50 ng·mL^−1^) for 1, 2, and 4 days. The results showed that the number of SA-β-gal positive cells was gradually increased over time and reached about 12 folds compared with that in controls at day 4 (Fig. 2a). Another common feature of senescent cells is growth arrest.^16^ We then performed cell counting kit 8 (CCK8) assay to determine how IL-13 affected cell proliferation. The proliferation ability of SMG-C6 was significantly decreased by 50, 100, and 150 ng·mL^−1^ IL-13 treatment for 4 days (Fig. 2b). Besides, 50 ng·mL^−1^ IL-13 obviously inhibited the cell proliferation from day 4, and this effect lasted to day 8 (Fig. 2c). Accordingly, we chose 50 ng·mL^−1^ as the drug concentration in the following experiments. It is well known that cellular senescence is primarily controlled by p53/p21 and p16 pathways, both of which inhibit cell cycle progression.^17^ IL-13 significantly upregulated the protein and mRNA expression of p53 and p16, but had no significant effect on p21 (Fig. 2d, e). We further measured the mRNA expression of SASP factors that can be secreted by the senescent cells.^8,9^ The mRNA level of IL-1β and IL-6 were markedly increased by IL-13 treatment for 4 days, whereas the mRNA level of TGF-β1 was not changed in SMG-C6 cells (Fig. 2f). These results altogether showed that IL-13 was the trigger that directly induced cellular senescence in salivary gland epithelial cells.

### Protein profile analysis shows that mitochondrial dysfunction and oxidation–reduction imbalance are involved in SMGs of IgG4-RS

**Fig. 3:**
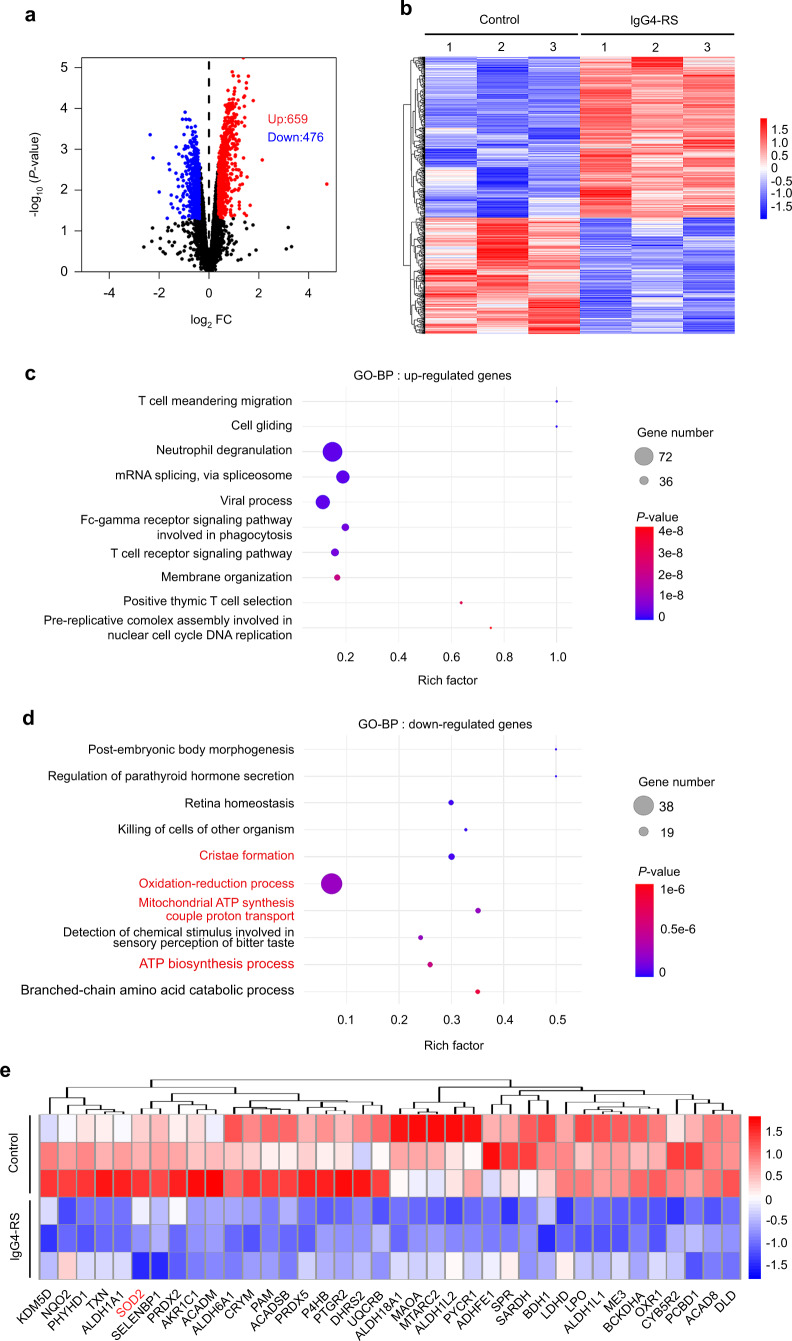
Protein profile analysis displays that mitochondrial dysfunction occurs in the SMGs of IgG4-RS patients. **a**, **b** Volcano plot (**a**) and heat map (**b**) showing 1 135 genes with |fold change| > 1.3 and *P* < 0.05 were found in the SMGs of IgG4-RS patients (*n* = 3) compared to controls (*n* = 3). **c**, **d** Top 10 of GO-BP analysis for up-regulated proteins (**c**) and down-regulated proteins (**d**) in the SMGs of IgG4-RS patients. **e** Heat map showing the down-regulated proteins under the term “oxidation–reduction process” in GO-BP-enriched categories

To explore the potential mechanism regarding the involvement of cellular senescence in the pathogenesis of IgG4-RS, we performed proteomics and bioinformatics of the SMG samples from IgG4-RS patients and controls. A total of 1 135 (659 up-regulated and 476 down-regulated) differentially expressed proteins (DEPs) were identified in IgG4-RS SMGs compared to controls (Fig. 3a, b). Gene ontology biological process (GO-BP) analysis revealed that the up-regulated DEPs in IgG4-RS were mostly associated with immune-related biologic processes, such as T cell meandering migration, T cell receptor signaling pathway, and positive thymic T cell selection (Fig. 3c), while the down-regulated DEPs were mainly enriched in mitochondrion-related biologic processes, such as oxidation–reduction process, cristae formation, mitochondrial ATP synthesis couple proton transport, and ATP biosynthesis process (Fig. 3d). Furthermore, we analyzed the down-regulated DEPs under the term “oxidation–reduction process” in GO-BP-enriched categories. Many redox reaction-related proteins, especially the antioxidant system such as superoxide dismutase 2 (SOD2), peroxiredoxin 5 (PRDX5), and thioredoxin (TXN), were significantly lower in IgG4-RS SMGs (Fig. 3e). These data hinted that mitochondrial dysfunction and oxidation–reduction imbalance might be involved in the pathogenesis of IgG4-RS.

### IL-13 causes mitochondrial dysfunction through increasing mitochondrial ROS (mtROS) accumulation

**Fig. 4:**
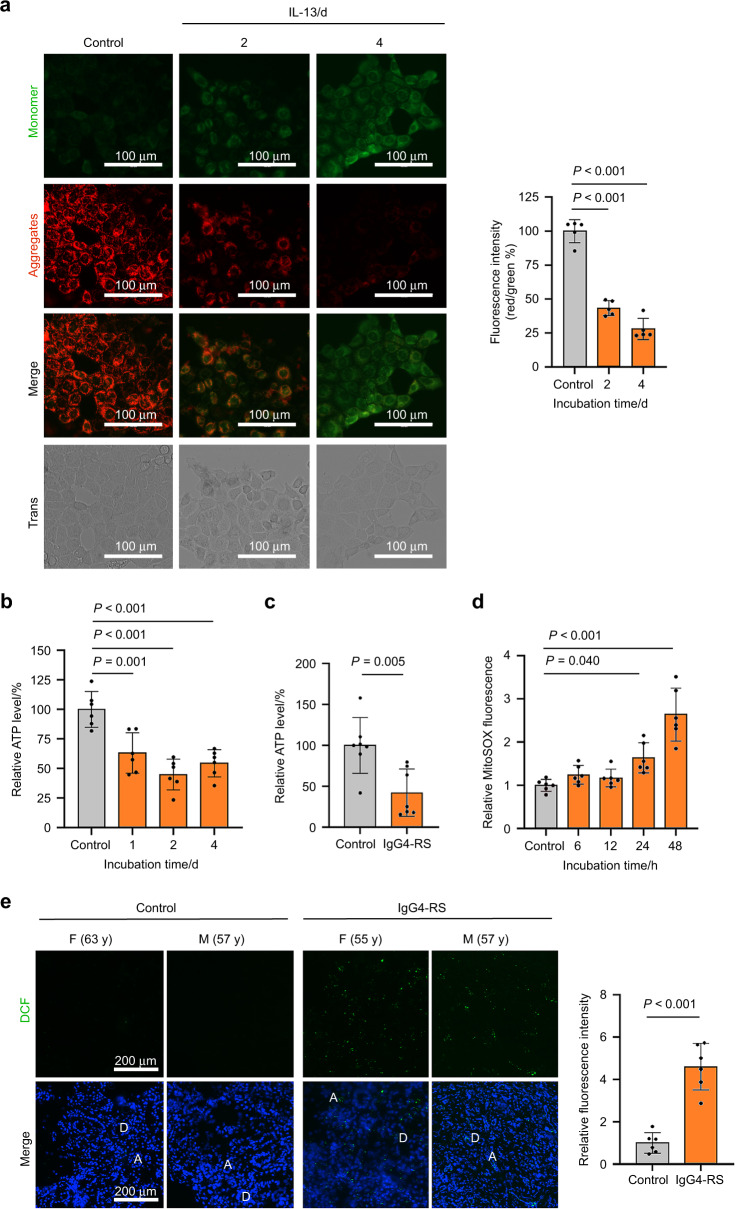
IL-13 promotes mitochondrial dysfunction through promoting mtROS accumulation. **a** Left: the MMP of SMG-C6 cells treated with IL-13 (50 ng·mL^−1^) for 2 and 4 days was measured via JC-1 staining (*n* = 5 per group). Up-right: quantitative analysis was measured by the ratios of red/green fluorescence. **b** The ATP level by treatment of IL-13 (50 ng·mL^−1^) for 1, 2 and 4 days was measured using firefly luciferase luminescence in SMG-C6 cells (*n* = 6 per group). **c** The level of ATP in the SMGs of IgG4-RS patients (*n* = 7) and controls (*n* = 7). **d** MitoSOX was used to detect the mtROS level after treatment with IL-13 (50 ng·mL^−1^) for 6, 12, 24, and 48 h in SMG-C6 cells (*n* = 6 per group). **e** The intracellular total ROS level (right) and quantitative analysis (left) in the SMGs of IgG4-RS patients (*n* = 6) and controls (*n* = 6) was detected by DCFH-DA staining. A acinus, D duct, M male, F female, y years old. The significance of differences between groups was analyzed by unpaired Student’s *t*-test (**c**, **e**) or one-way ANOVA followed by Bonferroni’s test (**a**, **b**, **d**). Data are presented as the mean ± SD

**Fig. 5:**
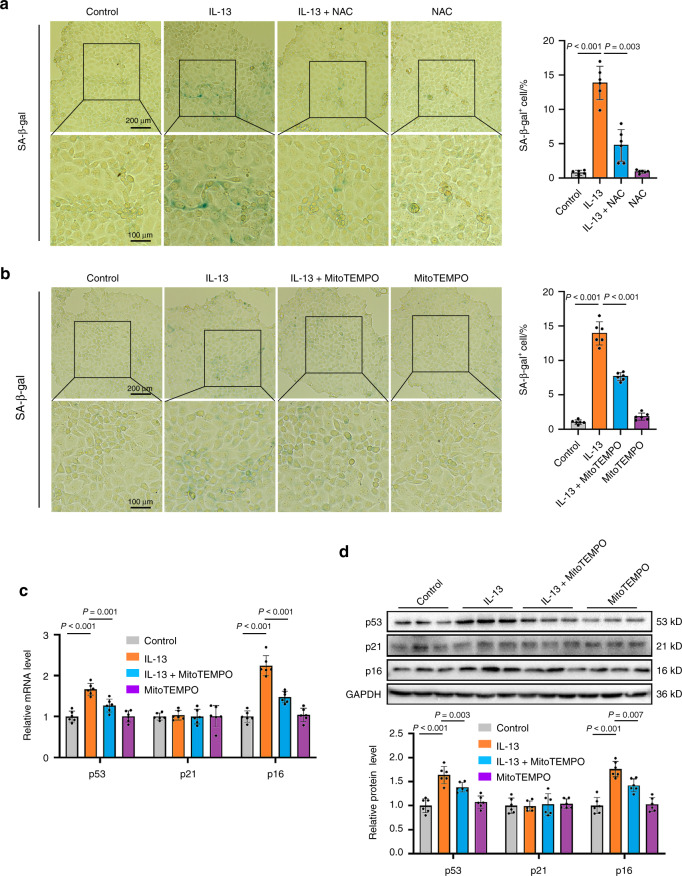
Pretreatment with reactive oxygen scavengers inhibits the IL-13-induced cellular senescence. **a**, **b** SMG-C6 cells were pretreated with NAC (10 μmol·L^−1^) (**a**) and MitoTEMPO (1 μmol·L^−1^) (**b**) for 30 min, and then incubated with IL-13 (50 ng·mL^−1^) for 4 days. The cellular senescence was measured by SA-β-gal staining (*n* = 6 per group). **c**, **d** SMG-C6 cells were pretreated with MitoTEMPO (1 μmol·L^−1^) for 30 min, and then incubated with IL-13 (50 ng·mL^−1^) for 4 days. The mRNA (**c**) and protein (**d**) expression of p53, p21, and p16 were detected by using quantitative real-time PCR and western blot, respectively (*n* = 6 per group). The significance of differences between groups was analyzed by one-way ANOVA followed by Bonferroni’s test. Data are presented as the mean ± SD

Since mitochondrial dysfunction has been identified as one of the main triggers of cellular senescence, we next tested whether the mitochondrial dysfunction was involved in the IL-13-induced cellular senescence. Mitochondrial membrane potential (MMP) is an indicator of mitochondrial function, and can be determined by using JC-1 which is a fluorescent dye sensitive to MMP. As shown in Fig. 4a, IL-13 treatment caused a gradual reduction in MMP as observed by decreased ratios of red/green fluorescence at day 2 and 4 in SMG-C6 cells. Besides, the cellular ATP content was significantly decreased as early as 1 day after IL-13 treatment (Fig. 4b), again suggesting that the mitochondrial function was impaired by IL-13. Moreover, the reduction of ATP level was also detectable in the SMG tissues of IgG4-RS patients (Fig. 4c). Excessive accumulation of mtROS is a well-known contributor to mitochondrial dysfunction.^18^ Here, we observed a significant increase in mtROS level after treatment with IL-13 for 24 h, and this increase became much more obvious in the cells treated with IL-13 for 48 h (Fig. 4d). The ROS level was significantly elevated in the residual acinar and ductal cells in the SMGs of IgG4-RS patients than that of controls (Fig. 4e). To determine whether the IL-13-induced mtROS accumulation and mitochondrial dysfunction could lead to cellular senescence, we used both N-acetyl-L-cysteine (NAC) and MitoTEMPO, the scavenger for total ROS and specific mtROS, respectively. Pretreatment with either NAC (10 μmol·L^−1^) or MitoTEMPO (1 μmol·L^−1^) significantly abolished the IL-13-induced increased proportion of SA-β-gal positive staining cells (Fig. 5a, b). Furthermore, MitoTEMPO pretreatment reduced the IL-13-induced elevated mRNA and protein expression of p53 and p16 (Fig. 5c, d). These above results indicated that IL-13 elevated mtROS content and caused mitochondrial dysfunction, thereby contributing to cellular senescence in salivary epithelial cells.

### Signal transducer and activator of transcription 6 (STAT6) serves as the intracellular signal that mediates the IL-13-induced cellular senescence

**Fig. 6:**
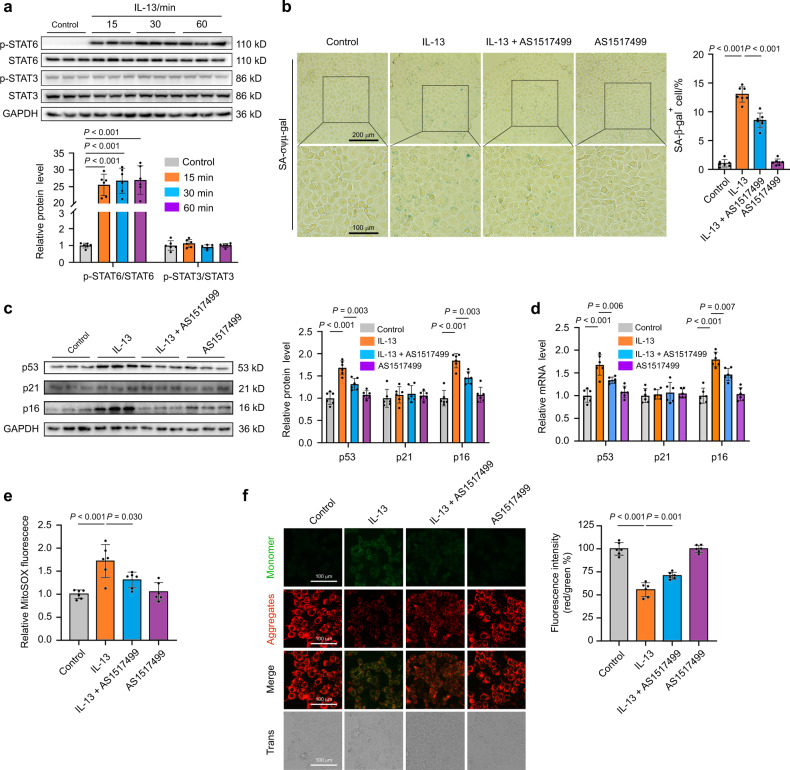
The STAT6–mtROS pathway mediates the IL-13-induced cellular senescence. **a** Upper: western blot was used to detect the phosphorylation of STAT6 and STAT3 in SMG-C6 cells treated with IL-13 (50 ng·mL^−1^) for 1, 2, and 4 days (*n* = 6 per group). Lower: quantification was normalized to total STAT6 or STAT3 (*n* = 6 per group). **b** SMG-C6 cells were pretreated with STAT6 inhibitor AS1517499 (100 nmol·L^−1^) for 30 min, and then incubated with IL-13 (50 ng·mL^−1^) for 4 days. The cellular senescence was measured by SA-β-gal staining (*n* = 7 per group). **c**, **d** SMG-C6 cells were pretreated with AS1517499 (100 nmol·L^−1^) for 30 min, and then incubated with IL-13 (50 ng·mL^−1^) for 4 days. The protein (**c**) and mRNA (**d**) expression of p53, p21, and p16 were measured by western blot and quantitative real-time PCR, respectively (*n* = 6 per group). **e** MitoSOX was used to detect the mtROS level in SMG-C6 cells with pretreatment of AS1517499 (100 nmol·L^−1^) for 30 min, and then incubated with IL-13 (50 ng·mL^−1^) for 24 h (*n* = 6 per group). **f** Left: the mitochondrial membrane potential (MMP) was measured using JC-1 in SMG-C6 cells with pretreatment of AS1517499 (100 nmol·L^−1^) for 30 min, and then incubated with IL-13 (50 ng·mL^−1^) for 2 days. Right: quantitative analysis was measured by the ratios of red/green fluorescence (*n* = 6 per group). The significance of differences among groups was analyzed by one-way ANOVA followed by Bonferroni’s test. Data are presented as the mean ± SD

Furthermore, the intracellular signals that mediated IL-13-induced cellular senescence were explored. STAT3 and STAT6 are two key transcription factors that can be regulated by IL-13. Upon IL-13 stimulation, the phosphorylated STAT3 (p-STAT3) and/or STAT6 translocate into the nucleus and then activate the transcription of multiple target genes.^19^ Here, we found that IL-13 treatment for 15 min significantly promoted STAT6 phosphorylation at Tyr641, whereas no obvious change in STAT3 phosphorylation was detected (Fig. 6a). To determine whether STAT6 participated in the IL-13-induced cellular senescence, AS1517499, a potent STAT6 inhibitor was used. Pretreatment with AS1517499 (100 nmol·L^−1^) significantly reduced the IL-13-induced increased number of SA-β-gal-positive cells (Fig. 6b). The IL-13-induced increases in p53 and p16 protein and mRNA expression were also inhibited by AS1517499 (Fig. 6c, d). Furthermore, pretreatment with AS1517499 significantly attenuated the IL-13-induced mtROS accumulation and MMP decrease (Fig. 6e, f). These data suggested that IL-13-induced cellular senescence through STAT6–mtROS pathway in SMG-C6 cells.

### The decreased expression and activity of SOD2 is responsible for the IL-13-induced mtROS accumulation

**Fig. 7:**
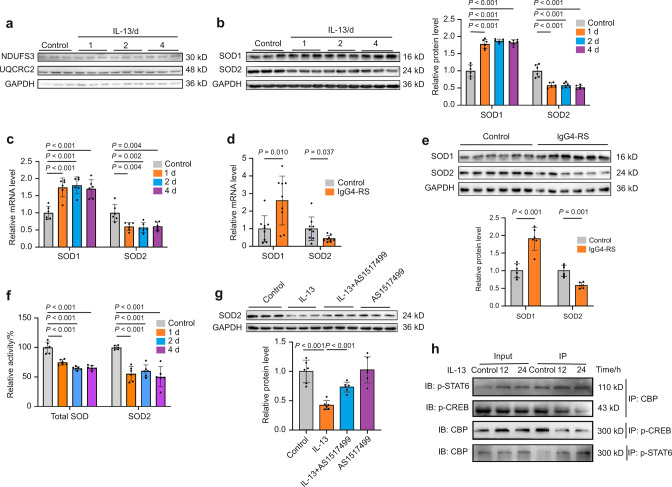
IL-13 decreases the expression and activity of SOD2 by decreasing the transcription activity of CREB. **a–c** SMG-C6 cells were incubated with IL-13 (50 ng·mL^−1^) for 1, 2, and 4 days. **a** Western blot analysis showing the expression of NDUFS3 and UQCRC2 proteins in SMG-C6 cells (*n* = 6 per group). **b** Western blot (left) and quantitative analysis (right) showing the expression of SOD1 and SOD2 proteins in SMG-C6 cells (*n* = 6 per group). **c** The mRNA expression of SOD1 and SOD2 were measured by quantitative real-time PCR in SMG-C6 cells (*n* = 6 per group). **d**, **e** Quantitative real-time PCR (**d**) (*n* = 9 per group) and western blot (**e**) (*n* = 6 per group) analysis showing the mRNA and protein expression of SOD1 and SOD2 in the SMGs of IgG4-RS patients and controls, respectively. **f** The activities of total SODs and SOD2 were detected in SMG-C6 cells treated with IL-13 (50 ng·mL^−1^) for 1, 2, and 4 days. Total SODs and SOD2 activities were normalized to the total protein content (*n* = 6 per group). **g** SMG-C6 cells were pretreated with AS1517499 (100 nmol·L^−1^) for 30 min, and then incubated with IL-13 (50 ng·mL^−1^) for 4 days. The protein expression of SOD2 was measured by western blot (*n* = 6 per group). **h** SMG-C6 cells were incubated with IL-13 (50 ng·mL^−1^) for 12 and 24 h. Co-IP and western blot analysis showing the interactions between CBP and p-STAT6 or p-CREB. The significance of differences between groups was analyzed by unpaired Student’s *t*-test (**d**, **e**) or one-way ANOVA followed by Bonferroni’s test (**b**, **c**, **f**, **g**). Data are presented as the mean ± SD

The accumulation of mtROS can be caused by increases in mtROS production and/or decreases in mtROS scavenger. Mitochondrial respiratory chain complexes, particularly complex I and III, are the main sites of mtROS production in cells.^20^ Our above proteomics results did not show any change in the protein level of complex I (nicotinamide adenine dinucleotide dehydrogenase ubiquinone Fe-S protein 3, NDUFS3) and complex III (ubiquinol-cytochrome C reductase complex core protein 2, UQCRC2) in the SMGs between IgG4-RS patients and controls. Consistently, the protein expression of NDUFS3 and UQCRC2 were not significantly altered by IL-13 treatment for 1, 2, and 4 days in SMG-C6 cells (Fig. 7a), suggesting that the elevated mtROS level might not be caused by its over-generation at the respiratory chain complexes. We next focused on the changes of the two important intracellular SODs. The protein and mRNA expression of SOD1 were increased whereas those of SOD2 were decreased by IL-13 treatment for 1, 2, and 4 days in SMG-C6 cells (Fig. 7b, c). In addition, mRNA and protein expression of SOD1 was higher together with a lower expression of SOD2 in the SMGs of IgG4-RS patients than those in controls (Fig. 7d, e). Moreover, it was notable that both total SODs and SOD2 activities were significantly decreased after IL-13 treatment for 1, 2, and 4 days (Fig. 7f). Then we explored whether SOD2 was the downstream target underlying STAT6. Pretreatment with AS1517499 significantly attenuated the IL-13-induced downregulation of SOD2 protein in SMG-C6 cells, whereas AS1517499 alone did not affect SOD2 expression (Fig. 7g). CAMP-response element binding protein (CREB) is a transcription factor that promotes the transcription of SOD2 as previously reported.^21^ The phosphorylation of CREB at Ser133 promotes its transcriptional activity by its interaction with the coactivator CREB-binding protein (CBP).^22^ Similarly, STAT6 also participated in transcriptional regulation by interacting with CBP.^23^ Co-immunoprecipitation (Co-IP) results showed that IL-13 treatment for 12 and 24 h induced an increased interaction between p-STAT6 and CBP, together with a decreased interaction between p-CREB and CBP, as evidenced in the immunoprecipitated proteins by the antibody against CBP, p-CREB or p-STAT6 (Fig. 7h). These results indicated that there might be a competitive relationship between p-STAT6 and p-CREB in regard to the combination with CBP. Hence, while IL-13 promoted the combination between p-STAT6 and CBP, the interaction between p-CREB and CBP was decreased, thereby resulting in a declined transcription activity of p-CREB and contributing to the reduced expression and activity of SOD2 in salivary gland epithelial cells.

## Discussion

**Fig. 8:**
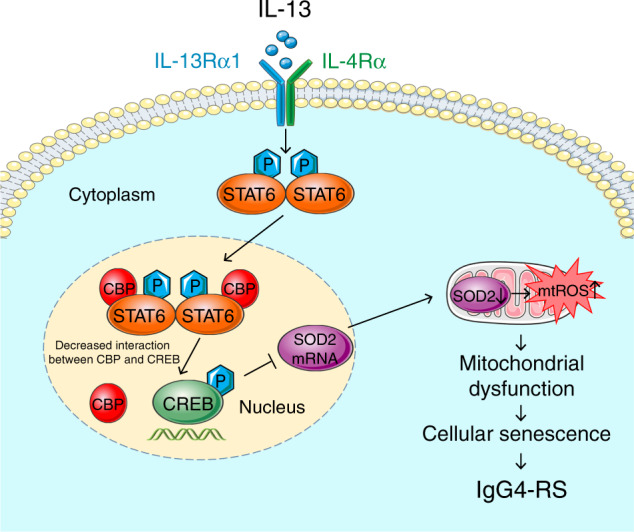
Schematic illustrates a proposed mechanism of IL-13-induced cellular senescence in salivary epithelial cells. The binding of IL-13 to its functional receptor, the IL-13Rα1/IL-4Rα heterodimer, leads to the phosphorylation of STAT6. The p-STAT6 monomers homodimerize and translocate to the nucleus and bind to CBP, which results in a decreased interaction between CREB and CBP and a decreased transcriptional activity of p-CREB. This would then cause the accumulation of mtROS by decreasing mRNA and protein expression of SOD2, thereby promoting mitochondrial dysfunction to induce cellular senescence

IgG4-RS is a recently recognized disease characterized by the infiltration of IgG4-positive plasma cells and fibrosis in the salivary glands along with elevated serum IgG4 levels. However, the pathogenesis of IgG4-RS is still unclear. Here, we found that the salivary gland acinar and ductal epithelial cells senescence occurred in IgG4-RS. Elevated IL-13 in the local lesions was an important cytokine to induce salivary gland epithelial cell senescence. Mechanistically, the accumulation of mtROS through STAT6–CREB–SOD2 signaling pathway contributed to the IL-13-induced senescence in salivary gland epithelial cells (a scheme was shown in Fig. 8). Our findings provided a novel insight of the pathogenesis of IgG4-RS.

Cellular senescence refers to a constant state of cell cycle arrest and produces multiple SASP cytokines, both of which impair the function of the affected organs and tissues and cause a variety of aging-related diseases. For example, cellular senescence leads to a higher production of pro-inflammatory cytokines and matrix degrading enzymes, which contributes to the development of osteoarthritis.^24^ Many cardiovascular diseases in aging are partially a consequence of the vascular dysfunction induced by the endothelial cell senescence.^25^ The senescent salivary gland stem cells exist in the parotid gland of the patients with primary Sjögren’s syndrome.^26^ The numbers of senescence-associated T cells are increased in aged mice and Sjögren syndrome model mice due to the overexpression of chemokine (CXCL13 or CXCL12) in salivary gland.^27^ These studies indicated that cellular senescence may be involved in immune inflammatory disease of salivary gland. However, whether cellular senescence was involved in the occurrence of IgG4-RS was still undetermined. Our results identified an obvious elevation of SA-β-gal-positive cells in both acini and ducts of IgG4-RS (Fig. 1a, b, d), suggesting that the epithelial cellular senescence existed and might be related with the dysfunction of salivary glands in IgG4-RS.

One of the main features of IgG4-RS is the accumulation of IgG4^+^ plasma cells in the salivary glands. Many previous studies have shown that Th2 cells, which can be activated by environmental allergens and infectious pathogens, play a dominant role in the pathogenesis of IgG4-RD, and the Th2-produced cytokines including IL-13 are significantly elevated in the affected tissues in IgG4-RD patients.^1,28^ It is thought that in IgG4-RD these Th2 cytokines activate B cells to class switch from IgM to IgE and/or IgG4, thus resulting in the infiltration of IgG4^+^ plasma cells.^29^ In accordance with the previous study,^6^ we found that IL-13 was higher in the SMG tissues of patients with IgG4-RS (Fig. 1a, c, f). However, there was no significant change in the serum level of IL-13 between IgG4-RS and controls (Fig. 1g), suggesting that IL-13 might derive from and act on the local lesions rather than circulating system. IL-13 activates the intracellular downstream signaling molecules by binding with IL-13Rα1/IL-4Rα heterodimer.^30^ We also observed an increased IL-13Rα1 expression in the SMGs of IgG4-RS patients (Fig. 1d, e, f). These data indicated that IL-13 signal was activated in the local lesions and its role in the pathogenesis of IgG4-RS was worth further investigating.

Nowadays, the role of cytokine-induced senescence in human diseases has received much attention. Previous studies have reported that the cytokine-induced senescence serves as part of immune surveillance of tumors, and the application of interferons in the treatment of tumors through induction of cellular senescence is currently under investigation.^31,32^ Here, our data showed that IL-13 stimulation significantly increased SA-β-gal activity, decreased cell proliferation, and upregulated the expression of senescence markers and SASP factors in SMG-C6 cells (Fig. 2). These data suggested that the elevated IL-13 in local lesions could directly lead to salivary gland epithelial cell senescence. Importantly, it is worth noting the differences in pathogenetic mechanisms between IgG-RS and other autoimmune diseases. For example, in Sjögren syndrome which is dominated by Th1 reactions, serum IL-13 level is significantly increased in patients, and IL-13 is found to aggravate lacrimal gland destruction and dysfunction by promoting the production of interferon-γ as well as the recruitment of mast cells into the glands.^5^ However, till now, there is no report showing whether IL-13 contributed to cell senescence in Sjögren syndrome. Therefore, IL-13 might play a different role in the pathogenesis of these two diseases. Considering that eliminating the IL-13-producing cells or neutralizing IL-13 is reported to improve salivary gland function in a mouse model of Sjögren syndrome,^33^ targeting IL-13 might be a promising therapeutic strategy for treatment of IgG4-RS through modulating the progression of cellular senescence.

Next, we explored the mechanism that is involved in the IL-13-induced cellular senescence in salivary gland epithelial cells. ROS are considered to be a crucial causative factor of cellular senescence and aging.^34^ ROS are mainly produced by mitochondrial respiratory chain, which is why mitochondria are the primary targets of oxidative damage, and a decline in mitochondrial function will in turn increase ROS production. Emerging studies have revealed a close relationship between mitochondrial dysfunction and many kinds of age-related diseases.^35,36^ But whether this phenomenon existed in IgG4-RS was totally unknown. We found that IL-13 promoted mtROS accumulation and caused decreases in both ATP and MMP (Fig. 4a, b, d), whereas pretreatment with antioxidants, especially for mtROS, inhibited the IL-13-induced cellular senescence (Fig. 5a-d). These results indicated that mitochondrial dysfunction induced by increased mtROS was responsible for the IL-13-induced cellular senescence in salivary gland epithelial cells.

Furthermore, the intracellular signaling pathway connecting IL-13 and mtROS was explored. As a canonical downstream molecule, STAT6 was obviously activated by IL-13 in SMG-C6 cells (Fig. 6a). Pretreatment with STAT6 inhibitor significantly ameliorated the IL-13-induced mtROS accumulation and MMP decrease as well as cellular senescence (Fig. 6b–f). Indeed, the application of ROS and STAT6 inhibitors did partially but not completely inhibit the IL-13-induced responses, suggesting that there might be another molecules/signaling pathway involved in this process. Gefitinib, a small molecule inhibitor of epidermal growth factor receptor (EGFR), significantly inhibits the IL-13-induced cellular senescence in IMR90 and HUVECs.^12^ In addition, IL-13 facilitates oxidative stress-induced cell death through activating janus kinase (JAK)/STAT6 and phosphoinositide-3-kinase/the mammalian target of rapamycin (PI3K/mTOR) pathway in both mouse and human dopaminergic neurons.^37^ These studies suggested that EGFR and PI3K-mTOR signal pathway may be other possible mechanisms underlying IL-13.

Our results showed that the protein expression of respiratory chain complex I and III, the primary sources of mtROS production, were unchanged by IL-13 stimulation (Fig. 7a). Besides, the protein level of these two complexes were not significantly altered in the SMGs of IgG4-RS patients. We then focused on the scavenge route of ROS through antioxidant defense. SODs are a group of an endogenous protective system that prevents oxidative injury by maintaining antioxidant defence. Three typical members are existed in the cytosol and the mitochondrial intermembrane space (Cu, Zn-SOD or SOD1), the mitochondrial matrix and inner membrane (Mn-SOD or SOD2), and extracellular compartment (extracellular SOD or SOD3).^38^ It should be noteworthy that SOD genes exhibit a high degree of homology between SOD1 and SOD3, with extremely low homology with SOD2,^39^ thus indicating that the transcription process might differ between SOD1/3 and SOD2. Besides, the antioxidant function of SOD2 in the mitochondrial matrix cannot be replaced by the presence or even overexpression of SOD1 in the cytosol, even though the antioxidant mechanism of SOD1 and SOD2 is similar,^40^ which emphasizes that SOD2 is irreplaceable for maintaining mitochondrial and cellular homeostasis. In the present study, the mRNA and protein expression of SOD2 were significantly reduced in both the SMGs of IgG4-RS patients and the IL-13-treated SMG-C6 cells (Fig. 7b-e). Although the SOD1 level was elevated at the same time, yet the activity of total SODs was still significantly decreased by IL-13 treatment (Fig. 7f). The results suggested that mtROS accumulation induced by IL-13 might mainly be due to the deficiency of SOD2 expression and function, which could not be compensated by the elevated SOD1 expression. Herein, these above data indicated that the impairment of mitochondrial scavenging system was an important cause of IL-13-induced redox imbalance in SMGs.

Finally, the possible regulation of SOD2 expression by IL-13 was investigated. Upon combination with CBP, CREB is a transcription factor that has been identified to directly upregulate the SOD2 expression.^21^ By contrast, no CREB binding site in SOD1 was found or reported in the previous studies.^41^ Since STAT6 also needs to be combined with CBP to promote its transcriptional activity,^23^ we therefore detected the interaction between CBP with either p-STAT6 or p-CREB. Co-IP assay showed an increased interaction between p-STAT6 and CBP, together with a decreased interaction between p-CREB and CBP (Fig. 7h), suggesting that there might be a competitive action between p-STAT6 and p-CREB to bind with CBP. Thus, the decreased transcriptional activity of p-CREB might explain the downregulation of SOD2 mRNA and protein expression induced by IL-13 in salivary gland epithelial cells. However, much work should be done to prove this speculation in our following studies.

In conclusion, our data revealed that IL-13 increased mtROS accumulation in a STAT6–CREB–SOD2-dependent manner, thereby contributing to the mitochondrial dysfunction and cellular senescence in salivary gland epithelial cells. These findings not only revealed a critical role of IL-13 in the cellular senescence through the induction of mitochondrial oxidative stress, but also provided potential therapies targeting IL-13 and mtROS-related cellular senescence for IgG4-RS.

## Materials and methods

### Patients and samples

According to the comprehensive diagnostic criteria, human SMG tissues were collected from the patients in Peking University Hospital of Stomatology who were diagnosed with IgG4-RS during September 2020 and October 2021.^42^ Supplementary Table 1 shows the clinical characteristics of the patients. Control SMG tissues, which were pathologically confirmed normal, were obtained from the age-matched individuals who had undergone neck dissection for head–neck cancers. Fresh tissue samples were immediately put into liquid nitrogen after surgery. Moreover, the SMG tissues from six patients with CS induced by calculi were used as disease controls (mean age = 55.5 ± 4.81; three males and three females). All patients had signed their informed consent prior to sample collection. This study was approved by the Ethics Committee of Peking University School and Hospital of Stomatology (No. PKUSSORB-2013008).

### Cell culture

Rat SMG epithelial polarized cell line SMG-C6 (a gift from Prof. David O. Quissell) was cultured at 37 °C with 5% CO_2_ in Dulbecco’s modified Eagle’s medium/nutrient mixture F-12 (DMEM/F-12) containing the following constituents: fetal bovine serum (2.5%), transferrin (5 mg·L^−1^), retinoic acid (0.1 μmol·L^−1^), glutamine (5 mol·L^−1^), thyronine T3 (2 nmol·L^−1^), insulin (5 mg·L^−1^), epidermal growth factor (80 μg·L^−1^), hydrocortisone (1.1 μmol·L^−1^), gentamicin sulfate (50 mg·L^−1^), penicillin (100 U·mL^−1^), and streptomycin (100 mg·L^−1^).

### Serum IL-13 level detection

A cytokine array was used to measure the level of IL-13 in the serum of eight IgG4-RS patients and eight controls, following the manufacturer’s instructions (Human Th1/Th2/Th17 Array Q1, QAH-TH17-1; RayBiotech).

### Protein profile analysis

The SMG tissues from three IgG4-RS patients and three age-matched controls were detected by proteomics analysis. The tissue processing was performed as described in our previous study.^43^ Briefly, we homogenized the SMG tissues on ice and used 100 μg of homogenates per sample for proteomics screening. After lysis, the concentration of protein was quantified by the Bradford assay. Then, the samples were digested overnight in trypsin at 37 °C and labeled with iTRAQ reagents according to the manufacturer’s instructions (AB Sciex). The labeled samples were analyzed using an EASY-Spray analytical column (120 mm × 75 μm, 3 μm) on an EASY-nLC1000 connected to a Q Exactive mass spectrometer (Thermo Fisher Scientific) and liquid chromatography–tandem mass spectrometry.

Analysis on the DEPs between IgG4-RS and control tissues was conducted with the edgeR package in R software. Data were standardized by log2 conversion. Specific screening conditions were followed by |fold change| >1.3 and *P* < 0.05. To dig into the potential function of these DEPs in SMGs, we precisely used the weighted enrichment analysis tools (WEAT) for the annotation of weighted gene function and pathway analysis through selecting “salivary gland” gene essentially score according to the instructions (http://www.cuilab.cn/weat/).^44^ GO-BP analysis were further used to assess the weighted DEPs.

### SA-β-gal staining

The senescent cells in human SMG tissues were stained for SA-β-gal according to the manufacturer’s instructions (SG03; DOJINDO). In brief, each section (7 μm) was fixed in 4% paraformaldehyde for 3 min at room temperature and washed with phosphate buffered saline (PBS) for 3 times. SPiDER-β-gal working solution (pH 6.0) was added and incubated for 30 min at 37 °C. After washing with PBS for 3 times, the slices were examined under a laser confocal microscope (Leica TCS SP8. Excitation wave: 488 nm; emission wave: 561 nm). The five different fields were randomly selected from the same sample and the average intensity was measured as *n* = 1. And we have detected six samples from six IgG-RS patients (*n* = 6).

In SMG-C6 cells, the senescent cells were stained for SA-β-gal according to the manufacturer’s instructions (9860; Cell Signaling Technology). SMG-C6 cells (2 000 per well) were seeded in 12-well plate and cultured 24 h before ready for IL-13 (50 ng·mL^−1^) stimulation for 1, 2, and 4 days. Briefly, the cells were fixed with the fixative solution, and then incubated with β-galactosidase staining solution (pH 6.0) overnight at 37 °C. After washing with PBS for 3 times, the cells were examined under a light microscope (Q550CW; Leica), and SA-β-gal positive cells were stained as green-colored cells. Five different fields from each sample were randomly selected for further analysis.

### Cell proliferation assay

The proliferation ability of SMG-C6 cells was performed by using the CCK8 according to the manufacturer’s instructions (CK04; DOJINDO). SMG-C6 cells (1 000 per well) were plated in 12-well plate, and after 24 h, cells were stimulated by different doses of IL-13 for the indicated times. CCK8 solution (10% CCK8 in serum-free DMEM medium) was added and incubated for 3 h at 37 °C. Then the optical density (OD) values were measured at 450 nm by an EnSpire Multilabel Plate Reader (PerkinElmer).

### Oxidative stress detection

In human SMG samples, the intracellular total ROS accumulation was detected by DCFH-DA (D6883; Sigma) staining. Briefly, the sections (7 μm) were incubated with the DCFH-DA solution (10 μmol·L^−1^) for 30 min at 37 °C in the dark, and then immediately observed under a confocal microscope (excitation wave: 490 nm; emission wave: 520 nm). Five randomly selected fields from each sample were used for further analysis.

To detect the content of mtROS in SMG-C6 cells, 50 000 cells per well were plated in six-well plate, and cells were incubated with IL-13 (50 ng·mL^−1^) for the indicated times. Then, the cells were incubated with MitoSOX working solution (5 μmol·L^−1^ in serum-free DMEM medium) for 10 min at 37 °C and protected from light before flow cytometry detection (excitation wave: 510 nm; emission wave: 580 nm).

### Quantitative real-time PCR analysis

Total RNAs were extracted from homogenized human SMG tissues and SMG-C6 cells using TRIzol (15596018; Invitrogen) according to the manufacturer’s instructions. cDNAs were synthesized by using the HiScript III RT Super Mix for qPCR (R323-01; Vazyme) and amplified using 2 × Real Star Green Fast Mixture (A301-10; GenStar) under a PikoReal Real-Time PCR System (Thermo Fisher Scientific). The ΔCt method using GAPDH as reference gene was used to quantify the results. Supplementary Table 2 showed the primers used in this study.

### Western blot analysis

Human SMG tissues and SMG-C6 cells were homogenized and lysed in cold RIPA buffer (89900; Thermo Fisher Scientific) containing protease inhibitors (Roche) for protein extraction, and the protein concentration was measured by using the BCA kit (P0012; Beyotime). Equal amounts of total proteins (20 µg) were mixed and dissolved in 5 × SDS–PAGE loading buffer (P1040; Solarbio) and heated to 100 °C for 10 min. After separation on 10% or 12% SDS–PAGE, the proteins were transferred to polyvinylidene difluoride membranes and blocked with 5% nonfat milk, before being immunoblotted overnight at 4 °C with the primary antibodies, including p53 (10442-1-AP; Proteintech), p21 (10355-1-AP; Proteintech), p16 (PA1-30670; Invitrogen), STAT6 (5397; Cell Signaling Technology), p-STAT6 (Tyr641) (56554; Cell Signaling Technology), STAT3 (9139; Cell Signaling Technology), p-STAT3 (Tyr705) (9145; Cell Signaling Technology), NDUFS3 (ab110246; Abcam), UQCRC2 (ab14745; Abcam), SOD1 (37385; Cell Signaling Technology), SOD2 (BS6734; Bioworld Technology), CREB (9197;Cell Signaling Technology), CBP (7389; Cell Signaling Technology), and p-CREB (Ser133) (9198; Cell Signaling Technology). Membranes were washed with PBST for three times before being incubated with horseradish peroxidase-conjugated secondary antibodies (Zhongshan Laboratories). Then, the bands were visualized by using enhanced chemiluminescence reagent (Thermo Fisher Scientific). Image J software was used to quantify the density of bands. The relative protein level was calculated by normalizing GAPDH protein level, and then the obtained value was compared with that of the control group.

### Immunofluorescence staining

Immunofluorescence staining was used to detect the distribution and expression of IL-13 and IL-13Rα1 in human SMG tissues. Briefly, the 4% paraformaldehyde-fixed tissues were blocked with 1% BSA. Thereafter, the sections were probed with the primary antibody against IL-13 (JES10-5A2; Invitrogen) or IL-13Rα1 (ab79277; Abcam) at 4 °C overnight. After incubation with secondary antibodies conjugated to Fluor-488, sections were captured using a laser confocal microscope (Leica TCS SP8. Excitation wave: 488 nm; emission wave: 561 nm). The five different fields were randomly selected from the same sample and the average intensity was measured as *n* = 1. And we have detected six samples from six IgG-RS patients (*n* = 6).

### Measurement of ATP content

ATP-Lite assay kit was used to measure ATP content of human SMG tissues and SMG-C6 cells (T007; Vigorous Biotechnology) according to the manufacturer’s instructions. The ATP content was normalized to protein concentration and the results were expressed as fold change compared with control group.

### Detection of MMP

The MMP was evaluated by using the JC-1 dye (65-0851-38; Invitrogen). The higher the MMP is, the more JC-1 aggregates form to appear a red fluorescence, in contrast to the JC-1 monomer that has a green fluorescence. Thus, the MMP was displayed by the change in the ratio between red and green fluorescence. SMG-C6 cells (2 000 per well) were seeded in 12-well plate 24 h before stimulation with IL-13 (50 ng·mL^−1^) for 2 and 4 days. Then the cells were collected and stained with JC-1 for 30 min at 37 °C in the dark, and then determined by using fluorescence microscopy.

### Total SODs and SOD2 activity assay

SMG-C6 cells (2 000 per well) were seeded in 12-well plate and cultured for 24 h, and then stimulated with IL-13 (50 ng·mL^−1^) for 1, 2, and 4 days. The total SODs activity was measured by using the total superoxide dismutase assay kit with WST-8 (S0101M; Beyotime) according to the manufacturer’s instructions. To further detect the activity of SOD2, zinc diethyldithiocarbamate (1 mmol·L^−1^) was added into the sample to inhibit the activity of SOD1 and extracellular SOD3 as previously reported.^45^ The absorbance at 450 nm was measured. Total SODs and SOD2 activities were normalized to the total protein content. The results were expressed as fold change compared with control group.

### Co-IP assay

Co-IP assay was performed to determine the interactions between two interested proteins in SMG-C6 cells. In brief, the cells were collected and washed with PBS. Then, SMG-C6 cells were lysed with RIPA buffer (89900; Thermo Fisher Scientific) and harvested by centrifugation at 12 000 r·min^−1^ for 20 min. Subsequently, the cell lysates were incubated with Protein A/G Plus-Agarose beads (sc-2003; Santa Cruz Biotechnology) at 4 °C for 3 h. A portion of the cell lysates was removed as input control. Furthermore, the cell lysates were incubated with the Protein A/G Plus-Agarose beads and specific primary antibody at 4 °C overnight. Immunoprecipitates were collected and washed with cold PBS for 3 times. Finally, the immunoprecipitates and input controls were analyzed by western blot assay using the corresponding antibodies of interested proteins.

### Statistical analysis

All data are expressed as the mean ± standard deviation (SD). The significance of differences between groups was analyzed by unpaired Student’s *t*-test between two groups or one-way ANOVA followed by Bonferroni’s test among three and more than three groups. Correlation was analyzed by using Spearman test. All statistical analyses were performed by using SPSS 26.0. *P* < 0.05 was considered statistically significant.

## Supplementary information

Supplementary Materials
