# Supplementary material for: Interleukin-13 promotes cellular senescence through inducing mitochondrial dysfunction in IgG4-related sialadenitis
Source: Int J Oral Sci. 2022 Jun 20;14:29. doi: 10.1038/s41368-022-00180-6 (PMC9207030; doi:10.1038/s41368-022-00180-6)
Supplement: Supplementary file 1 — Supplementary Materials [file 41368_2022_180_MOESM1_ESM.doc]

**Supplementary Table1. Clinical characteristics and serologic examination of patients with IgG4-related sialadenitis (IgG4-RS).**

| **No.** | **Age** | **Sex** | **Swollen glands** | | | | **Systemic diseases** | **Serologic examination** | | | | |
| --- | --- | --- | --- | --- | --- | --- | --- | --- | --- | --- | --- | --- |
|  |  |  | **SMG** | **PG** | **SLG** | **LG** |  | **IgG4 (g/L)** | **IgG (g/L)** | **IgE (KU/L)** | **SS-A/Ro** | **SS-B/La** |
| 1 | 47 | M | + | - | - | - | Diabetes, CHD | 5.79 | 15.82 | 1208 | - | - |
| 2 | 68 | M | + | - | - | - | Diabetes, CHD | 13 | 21.65 | 110 | - | - |
| 3 | 49 | F | + | - | - | - | - | 3.17 | 16.47 | 253.6 | - | - |
| 4 | 58 | M | + | - | - | + | Hypertension | 5.87 | 26.2 | 237.4 | - | - |
| 5 | 63 | F | + | - | - | - | - | 3.6 | 13.46 | 359 | - | - |
| 6 | 50 | M | + | - | - | - | - | 2.98 | 13.78 | 127 | - | - |
| 7 | 57 | F | + | - | - | - | - | 1.98 | 13.24 | 134 | - | - |
| 8 | 55 | F | + | - | + | + | - | 25.3 | 24.14 | 652 | - | - |
| 9 | 58 | M | + | + | - | + | - | 25.7 | 25.2 | 835 | - | - |
| 10 | 49 | F | + | - | + | + | Hypertension, diabetes | 9.31 | 21.68 | 224.4 | - | - |
| 11 | 55 | F | + | - | + | + | - | 4.58 | 17.9 | 83.24 | - | - |
| 12 | 57 | M | + | - | + | + | Hypertension, diabetes | 4.38 | N/A | 1140 | - | - |

CHD, coronary heart disease. F, female. LG, lachrymal gland. M, male. N/A, not available. PG, parotid gland. SLG, sublingual gland. SMG, submandibular gland. +, positive; −, negative.

**Supplementary Table 2. List of real-time PCR primers.**

| **Gene** | **Upper primer (5’-3’)** | **Lower primer (5’-3’)** |
| --- | --- | --- |
| **Human** |  |  |
| IL-13 | GGTCATTGCTCTCACTTGCC | CAGCCTGACACGTTGATCAG |
| IL-13Rα1 | GTCCCTGGTGTTCTTCCTGATAC | CAGCACTACAGAGTCGGTTTC |
| SOD1 | GGCAAAGGTGGAAATGAAGA | GGGCCTCAGACTACATCCAA |
| SOD2 | GCTCATGCTTGAGACCCAAT | CACCCGATCTCGACTGATTT |
| GAPDH | ACATCATCCCTGCCTCTACTG | CCTGCTTCACCACCTTCTTG |
| **Rat** |  |  |
| p16 | TGCAGATAGACTAGCCAGGGGA | CTTCCAGCAGTGCCCGCA |
| p53 | GGACGACAGGCAGACTTTTC | TTTTATGGCGGGACGTAGAC |
| p21 | CCGAGAACGGTGGAACTTTGAC | GAACACGCTCCCAGACGTAGTTG |
| IL-1β | AATCTCACAGCAGCATCTC | ACACTAGCAGGTCGTCAT |
| IL-6 | GCTCTGGTCTTCTGGAGTTCC | GAGTTGGATGGTCTTGGTCCT |
| TGF-β1 | AGAAGTCACCCGCGTGCTAA | TCCCGAATGTCTGACGTATTGA |
| SOD1 | TTCGAGCAGAAGGCAAGCGGTGAA | AATCCCAATCACACCACAAGCCAA |
| SOD2 | GAGGCTATCAAGCGTGACTTTG | GCAATGGGTCCTGATTAGAGC |
| GAPDH | GTAAGAAACCCTGGACCACC | ACACTGCATTCACACACAAG |

IL, interleukin; IL-13Rα1, IL-13 receptor α1; SOD, superoxide dismutase; TGF-β1, transforming growth factor-β1.
